# Long-Term Stability of Gradient Characteristics Warrants Model-Based Correction of Diffusion Weighting Bias

**DOI:** 10.3390/tomography8010030

**Published:** 2022-02-04

**Authors:** Yuxi Pang, Dariya I. Malyarenko, Lisa J. Wilmes, Ajit Devaraj, Ek T. Tan, Luca Marinelli, Axel vom Endt, Johannes Peeters, Michael A. Jacobs, David C. Newitt, Thomas L. Chenevert

**Affiliations:** 1 Department of Radiology, University of Michigan, Ann Arbor, MI 48109, USA; tlchenev@med.umich.edu; 2 Department of Radiology & Biomedical Imaging, University of California, San Francisco, CA 94158, USA; lisa.wilmes@ucsf.edu (L.J.W.); dnewitt@sbcglobal.net (D.C.N.); 3 Philips Research Laboratories, Cambridge, MA 02141, USA; ajit.devaraj@philips.com; 4 Radiology and Imaging, Hospital for Special Surgery, New York, NY 10021, USA; tane@HSS.EDU; 5 GE Global Research, Niskayuna, NY 12309, USA; marinell@research.ge.com; 6 Siemens Healthcare GmbH, 91052 Erlangen, Germany; axel.vomendt@siemens-healthineers.com; 7 Philips MR Clinical Science, 5684 PC Best, The Netherlands; hans.peeters@philips.com; 8 Radiology and Radiological Science, Johns Hopkins University School of Medicine, Baltimore, MD 21205, USA; mikej@mri.jhu.edu

**Keywords:** diffusion weighted imaging (DWI), gradient nonlinearity (GNL), apparent diffusion coefficient (ADC), longitudinal multi-platform ADC QC studies, root-mean-square difference (RMSD)

## Abstract

The study aims to test the long-term stability of gradient characteristics for model-based correction of diffusion weighting (DW) bias in an apparent diffusion coefficient (ADC) for multisite imaging trials. Single spin echo (SSE) DWI of a long-tube ice-water phantom was acquired quarterly on six MR scanners over two years for individual diffusion gradient channels, along with $B_{0}$ mapping, as a function of right-left (RL) and superior-inferior (SI) offsets from the isocenter. Additional double spin-echo (DSE) DWI was performed on two systems. The offset dependences of derived ADC were fit to 4th-order polynomials. Chronic shim gradients were measured from spatial derivatives of $B_{0}$ maps along the tube direction. Gradient nonlinearity (GNL) was modeled using vendor-provided gradient field descriptions. Deviations were quantified by root-mean-square differences (RMSD), normalized to reference ice-water ADC, between the model and reference ($RMSD_{REF}$), measurement and model ($RMSD_{EXP}$), and temporal measurement variations ($RMSD_{TMP}$). Average $RMSD_{REF}$ was 4.9 ± 3.2 (%RL) and –14.8 ± 3.8 (%SI), and threefold larger than $RMSD_{EXP}$. $RMSD_{TMP}$ was close to measurement errors (~3%). GNL-induced bias across gradient systems varied up to 20%, while deviation from the model accounted at most for 6.5%, and temporal variation for less than 3% of ADC reproducibility error. Higher SSE $RMSD_{EXP}$ = 7.5–11% was reduced to 2.5–4.8% by DSE, consistent with the eddy current origin. Measured chronic shim gradients below 0.1 mT/m had a minor contribution to ADC bias. The demonstrated long-term stability of spatial ADC profiles and consistency with system GNL models justifies retrospective and prospective DW bias correction based on system gradient design models. Residual errors due to eddy currents and shim gradients should be corrected independent of GNL.

## 1. Introduction

In clinical MR diffusion weighed imaging (DWI), gradient nonlinearity (GNL) leads to spatially varying diffusion weighting [1] that causes predictable systematic errors or biases in derived metrics, such as an apparent diffusion coefficient (ADC) [2,3], a promising quantitative biomarker for cancer therapy response and diagnosis [4,5,6]. ADC is calculated based on a mono-exponential decay model for a DWI signal with increasing diffusion weighting quantified by the *b*-value. Since GNL induces a spatially nonuniform *b*-value with respect to the magnet isocenter [1,2,3], it will primarily compromise ADC measured over a large field-of-view (FOV) or for off-centered anatomy [6,7,8]. When DWI is employed in a multi-center study on multiple platforms, additional variability will confound ADC measurements due to different system-specific spatial GNL patterns [9,10,11,12]. To establish reliable diagnostic thresholds, efforts are underway to correct for this systematic variability either retrospectively via centralized data analysis [11,13] or prospectively, on systems equipped with vendor implementation of GNL bias correction [7,14].

The most practical correction of GNL-induced *b*-value bias would be to use scanner-specific gradient design information [1,2,3] in analogy to the current mitigation of geometric image distortions [15,16]. This approach relies on the assumption of high consistency between the static gradient system model and the observed biases, e.g., recently shown for several gradient systems over moderate (cranial) FOV [14,17]. To confirm feasibility of model-based GNL correction for both retrospective and prospective applications over arbitrary FOV in a multi-center, multi-scanner setting, our team has designed long-term DWI quality control studies using a quantitative ice-water diffusion phantom [18]. This phantom was scanned quarterly over two years on multiple representative clinical gradient platforms within typical body DWI FOV [5,6,8]. The described studies were performed in collaboration with three key MRI vendors, the participants of an academic industrial partnership (AIP).

The presence of eddy currents (EC) and object dependent $B_{0}$ shim variations may create local gradient fields, resulting in finite deviations of observed ADC bias from GNL models [2,10,19]. The longitudinal evaluation of spatially dependent diffusion weighting bias for individual gradient channels, with respect to vendor-provided gradient GNL models, would allow a quantitative assessment of non-GNL bias contributions. With the goal to inform an appropriate ADC bias correction strategy, this work evaluated the long-term stability of the systematic bias measured on a temperature-controlled diffusion phantom and identified the physical origins of observed non-GNL errors. These findings could be transformed into a better strategy for DWI acquisition and post processing toward increased ADC accuracy and reproducibility in multi-center multi-platform clinical trials.

## 2. Materials and Methods

### 2.1. DWI Phantom

**Figure 1:**
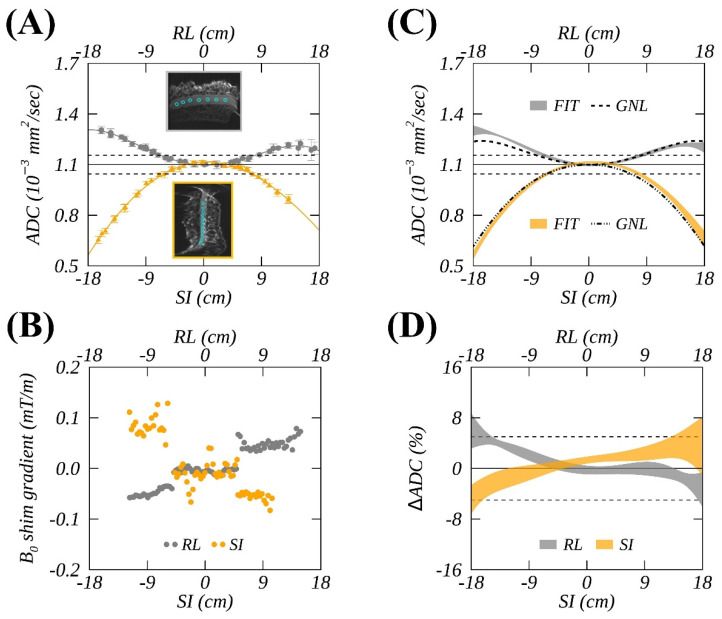
An example of ice-water spatial-dependent (i.e., right-left (RL) offsets in gray and superior-inferior (SI) offsets in orange) apparent diffusion coefficient (ADCs) measured for the $G_{y}$ gradient channel (**A**) and $B_{0}$ shim gradients (**B**) on a 1.5T MR scanner. Fitted ADCs (ribbons corresponding to temporal mean ± SD) from longitudinal studies were compared with a predicted (dashed curves) system-specific gradient nonlinearity (GNL) model in (**C**), and their relative differences normalized by an ADC reference (solid horizonal line) of 1.1 × 10^−3^ (mm^2^/s) are shown in (**D**). Mean ADC measurements within regions of interest (ROIs) (e.g., cyan circles in A-inset) are plotted as a function of RL and SI offsets, respectively, with error-bars corresponding to a standard deviation within an ROI. Dashed horizontal lines denote ± 5% deviations from the reference ice-water ADC value.

An ice-water phantom [18] and a customized positioning stage along with instructions (Supplementary Material S1 and S2) were shipped to three academic sites, the participants in an academic industrial partnership (AIP) project. The positioning stage allowed controlled phantom vertical locations (±y) and horizontal offsets along the ±z or ±x direction in the magnet reference frame. The phantom consisted of a 3.8-L plastic container with a central capped 175-mL measurement tube (2.9 cm diameter, 27 cm long), pre-filled with distilled water. About two hours before starting MR scans, the plastic container was filled with an ice water slurry and placed in an insulation foam sleeve so that the measurement tube reached thermal equilibrium and stayed at 0 °C during the exam, providing an apparent diffusion coefficient (ADC) of 1.10 (10^−3^ mm^2^/s) [20]. For all scanners, this ADC value was expected to be measured at a magnet isocenter, where magnetic field gradients are linear, and thus served as a universal reference (REF) value (Figure 1A,C,D, horizontal line) independent of scanner room temperature. 

### 2.2. MR Imaging Protocol

The ice-water phantom was scanned on six clinical scanners from three dominant MR vendors including both 3.0T and 1.5T platforms equipped with unique gradient systems, labelled as Sys1-6. For all systems, six test measurements were performed quarterly over a period of 20 months with unmodified gradient hardware. The SW upgrade happened only on Sys 4 for the last two test points. No other SW upgrades were confirmed in the submitted DICOM images. All studied MR systems were qualified for use in a clinical trial [6,7] except for Sys2. Conventional axial echo planar imaging (EPI) DWI were acquired using torso coil with the phantom measurement tube oriented along the x-axis or right-left (RL) direction and positioned at offsets of 0 and ±11 cm. These offsets helped to extend the spatial coverage as well as allowed the system to invoke multiple object-dependent shim conditions. Similarly, the phantom was scanned with the measurement tube along the z-axis or superior-inferior (SI) direction, with the same offsets as those used for axial images. Thus, the phantom was imaged at six positions in total between axial and sagittal scans (Supplementary Material S1 and S2). The field-of-view (FOV) and prescribed imaging slices remained centered at the isocenter for these six acquisitions while the “shim” volumes were offset and optimized accordingly using the best available system shim routines.

The key acquisition parameters were as follows (Supplementary Material S1 and S2): pulse sequence = single-shot spin-echo EPI using single echo (SSE) on all systems and additional double echo (DSE) on Sys1 and Sys5; FOV = 380 × 380 (mm^2^); acq/recon matrix = 200 × 200/256 × 256; number of slices = 15; slice thickness = 5 mm; TR/TE = at least 10 s/as short as possible (<150 ms); number of averages = 2; *b*-values = 0, 750, and 1500 (s/mm^2^); bandwidth/pixel (frequency-encoding) = 1500–2500 (Hz); and fat suppression was not used. Each *b* > 0 DWI was acquired using three diffusion gradients ($G_{x}$, $G_{y}$, $G_{z}$) applied along the primary magnet axes to characterize individual gradient channels.

In addition, $B_{0}$ mapping, based on phase difference from the multi-echo GRE sequence, were also obtained from the central slice along the phantom tube using the same shim settings as for DW imaging when the phantom was at six different positions. The measured imaging phase shifts were converted to resonance frequency offsets using a scan-specific echo interval (ΔTE = 2.5–5 ms); consequently, residual (or chronic) shim gradients were calculated (Figure 1B) as the spatial derivatives (along z and x directions) of these localized resonance frequency offsets. To assess the potential confounding effect of an incremental shim gradient on the ADC spatial bias, the spatial average shim gradients and their temporal variation were assessed for all systems.

The extent of shim-associated geometric distortions [21] was measured from an apparent phantom tube offset from a physical position along the phase-encode direction on the *b* = 0 image (up to 3 cm for Sys5 and Sys6). The separate DWI distortion from eddy currents [22] was assessed by relative compression of a phantom tube diameter for SSE versus DSE acquisition (available for Sys1 and Sys5) on the *b* = 1500 s/mm^2^ image along phase encode direction. No appreciable effect of concomitant fields [2] was expected for DSE using sagittal SI scans. An additional experiment with dial-in chronic shim gradient up to 0.1 mT/m was performed on Sys3 [23] to assess the maximum associated incremental impact on the ADC bias, for comparison to the measured chronic shim gradients on all the systems.

### 2.3. Spatial ADC Measurements

Following the mono-exponential diffusion decay model, ADC was calculated for individual gradient channels from DW images versus nominal b-value. While ADC was computed on a pixel-by-pixel basis, the mean ADC values were measured from multiple circular ROIs (~90 pixels) with an ~1 cm diameter. These ROIs were placed in the middle of the ice-water tube on the *b* = 0 image (Figure 1A, insets: cyan circles) while avoiding large susceptibility artifacts (e.g., near an air bubble or near edges) for signals free of severe geometric distortions. Typically, 20–30 offsets (ROIs) were identified for each phantom position, approximately covering a spatial extent from ±12 cm to ±17 cm. These produced about 50 non-overlapping ADC measurements along either the RL or SI direction for each of three gradient channels. Measurement uncertainty was estimated as 2 × SD (95% confidence interval [24]) of the ADCs within an ROI (Figure 1A, error bars), and was consistent with previous reports [10].

The measured spatial profile of the mean ADCs was then fit to a 4th-order polynomial model (Figure 1A, solid curves). For most gradient systems, a 2nd-order fit was indistinguishable from a 4th-order counterpart for ADC bias along the SI direction; however, a 4th-order fit was essential for profiles in the RL direction, particularly at larger offsets >10 cm. Therefore, the 4th-order fitting was performed for consistency for both RL and SI profiles from all gradient systems. Temporal variations of the fits acquired over two years were quantified by temporal standard deviation ($RMSD_{TMP}$) represented as the width of shaded ribbon plots (Figure 1C,D).

### 2.4. Theoretical Spatial ADC Based on GNL Model

Scanner-specific gradient nonlinearity tensors, L(r), were calculated numerically on a 4–5-mm 3D grid (spherical volume with a characteristic radius of 22.5–30.0 cm) within the magnet bores using gradient design information (i.e., spherical harmonics, SPH, coefficients) provided by the three MR vendors. Three-dimensional bias corrector maps for each gradient direction were generated as $C^{k}\left(r\right)=Tr\left(Lu_{k}Lu_{k}{}^{T}\right)\approx l_{kk}^{2}$ [3], where $u_{k}$ defined the direction cosines for three orthogonal DWI gradient directions along the primary magnet axes. These theoretical maps (normalized to 1 at the isocenter) were then multiplied by the reference value so that $C^{k}\left(0\right)$ becomes 1.10 (10^−3^ mm^2^/s). Spatially different theoretical ADCs were generated at various ROIs locations (e.g., Figure 1C, dashed curves) to which the measured spatial ADCs were compared. The measured locations were inferred from DICOM tags, and table offsets were accounted for if existing: On Sys5 and Sys6, an inferior offset of 1.3 cm was present for all measurements; an anterior offset of 2.3 cm was detected for Sys6; and a superior offset of 2.5 cm was observed for Sys1 and 2 for a single time point.

### 2.5. Data Analysis

The comparison of theoretical (GNL model-based) ADC profiles to measurement fit was performed over the range of ±18 cm to avoid excessive extrapolation of the fit to the measured data (typically, available for up to 12- to 17-cm offsets). Both fitted (“FIT”) and model (“GNL”) ADC spatial profiles were resampled to the same 0.2-cm resolution within the comparison range along RL and SI directions. To quantify average spatial deviations, three percent root-mean-square difference metrics normalized to the ice-water reference ADC (REF) were calculated, i.e., $\%RMSD\left(x,y\right)=\left(100\%/REF\right)\sqrt{\overset{N}{\underset{1}{\sum }}{\left(x-y\right)}^{2}/N}$, with N denoting the number of spatial ADC values.

The first metric, $\%RMSD_{REF}=$ %RMSD(GNL,REF), quantified the difference between the theoretical ADC bias and the reference (“GNL vs. REF”). The second, $\%RMSD_{EXP}=\%RMSD\left(GNL,mFIT\right)$, quantified the difference between the GNL model and the measured fit, with mFIT standing for the temporal mean of fits over *M* longitudinal measurements. The last metric, $\%RMSD_{TMP}=\overset{N}{\underset{1}{\sum }}\%RMSD\left(FIT,mFIT\right)/N$, quantified the spatial average of the temporal variation of the fits over the longitudinal studies (“Temp FIT”), with FIT denoting individual fits. These metrics were compiled for different ADCs from individual diffusion gradients and the trace (*x*, *y*, *z*, and *t*), i.e., $ADC_{t}=\underset{i}{\sum }ADC_{i}/3,\;i=x,y,z$.

Temporal variations were visualized by the ribbon plots (Figure 1C,D), where the ribbon edges represent the temporal mean ± standard deviation of measured ADCs at sampled spatial locations. Normalized by REF, the relative RMS differences between the measured and predicted ADCs are plotted in Figure 1D. The comparison across systems was summarized by RMSD median and ranges. The spatial mean of measured chronic shim gradients was expressed in %ADC error based on a previous simulation and a dialed-in de-shim calibration experiment [10,23]. Data analysis and visualization were performed with in-house scripts coded in the program languages of Matlab 2020b (The Mathworks, Inc., Natick, MA, USA) and IDL 8.8 (Harris Geospatial Solutions, Inc., Broomfield, CO, USA).

## 3. Results

### 3.1. Temporal Variations in Spatial ADC Measurements

**Figure 2:**
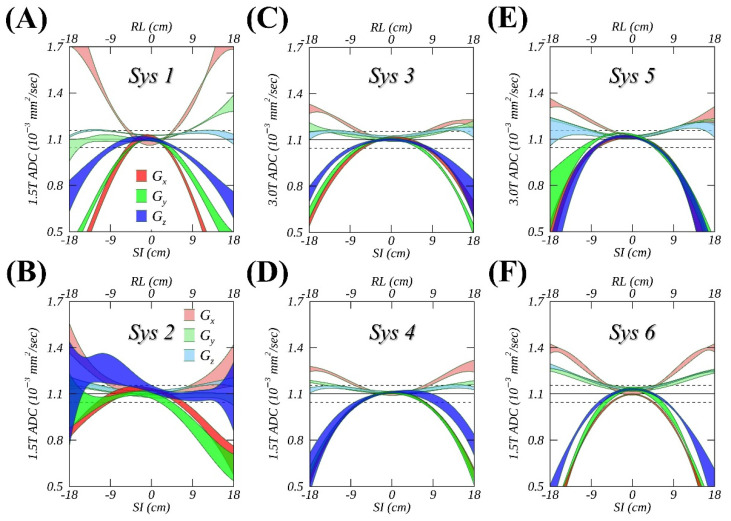
Spatial variations (colored ribbons representing temporal mean ± SD) of fitted ice-water apparent diffusion coefficient (ADCs) measured from longitudinal studies are shown as a function of a horizontal offset (light colors) and along the magnet bore (dark colors) for six different MR systems (Sys1-6) in (**A**–**F**), using three individual gradient channels $G_{x}$ (red), $G_{y}$ (green), and $G_{z}$ (blue). Solid and dashed horizonal lines mark an ice-water ADC reference and its ± 5% deviations.

The measured spatial ADC deviations from the reference, summarized in Figure 2 and Table 1 for gradient channels of all systems, mostly appeared either positive (RL) or negative (SI), consistent with the theoretical GNL models for the studied clinical MR systems (e.g., Figure 1C). The extent of measured absolute bias along SI was approximately twice of that along RL (e.g., Sys3-6), reaching about 50% near the FOV edges. The longitudinal variability reflected by ribbon width was consistent with the ROI measurement errors (e.g., Figure 1A, error bars), which is substantially smaller than an absolute deviation from the reference observed for most studied systems and gradient channels (except for Sys2, Figure 2B). Qualitatively, higher longitudinal consistency was observed for measurements closer to the isocenter (within 10 cm), and variability increased toward the FOV edges. This was mainly due to the lack of reliable measurements (fit extrapolation) for >10 cm offsets of several time points and proximity to susceptibility artifacts (edge EPI distortions).

From the same vendor, Sys1 (Figure 2A) considerably outperformed Sys2 (Figure 2B) at the same $B_{0}$ field strength (1.5T), suggesting an insufficient measurement accuracy for Sys 2. An asymmetric ADC profile, with right offsets observed for $G_{y}$ (light green) on Sys1, further revealed that non-GNL sources contributed a substantial (~10%) amount to the observed ADC spatial bias for this specific gradient channel. Overall, more channel-specific asymmetry (<5%) was observed for ADC profiles measured on 3T systems (Figure 2C,E) compared to 1.5T (Figure 2D,F), reflecting the fact that the observed incremental non-GNL bias could not be sufficiently mitigated even with higher (i.e., 2nd) order shims. Several systems also exhibited a minor (< 3%) constant shift of the fit at zero offset for the slice-select channel (e.g., Figure 2A,F, $G_{z}$ and $G_{y}$), which was consistent with finite cross-terms with imaging gradients.

### 3.2. ADC Measurements from SSE and DSE

**Figure 3:**
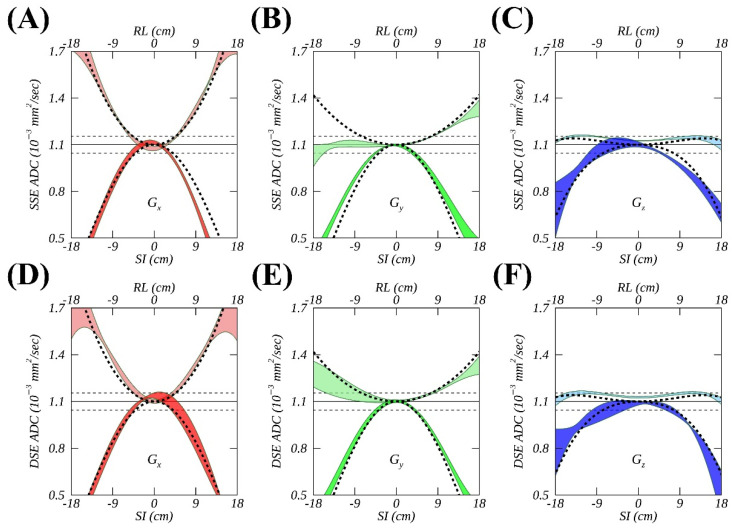
Temporal variations of spatial apparent diffusion coefficient (ADC) profiles (colored ribbons representing temporal mean ± SD) measured using single spin echo (**A**–**C**) and double spin echo (**D**–**F**) pulse sequences for a single MR system (Sys 1), compared to the gradient nonlinearity (GNL) model (dashed black curves) for three physical gradient channels, i.e., $G_{x}$ (red), $G_{y}$ (green), and $G_{z}$ (blue). Solid and dashed horizonal lines mark an ADC reference and its ± 5% deviations.

Figure 3 further highlights the differences in measured ADC fits on Sys1 when using SSE (Figure 3A–C) and DSE (Figure 3D–F) diffusion gradient sequences for $G_{x}$ (red), $G_{y}$ (green), or $G_{z}$ (blue). The RMS deviations between the “FIT” and “GNL” model (Figure 3, dashed curves) for $G_{y}$ with RL offsets visibly decreased to 3.2% from 8.3% when using DSE (Figure 3E) compared to SSE (Figure 3B) acquisitions. DSE reduced $\%RMSD_{EXP}$ to values closer to the measurement error observed for $G_{x}$ (1.9%) and $G_{z}$ (1.2%) versus SSE. Similar $\%RMSD_{EXP}$ improvements for DSE versus SSE were also observed on Sys5 for all three diffusion gradients, i.e., 3.3% vs. 7.6% ($G_{x}$), 2.6% vs. 4.1% ($G_{y}$), and 4.8% vs. 11.1% ($G_{z}$), with SI offsets. The inspection of corresponding phantom DWI images (not shown) indicated the presence of about 10% compression of the measurement tube diameter along phase encode direction for the SSE diffusion gradients, which were mitigated by DSE. These findings imply that eddy currents in SSE acquisitions were a likely source of the observed systematic ADC deviations from GNL models.

For systems and locations with good quality 1st-order shims (Sys1-Sys4), the EPI distortions in DW images led to <1 cm image distortions for z < 15 cm, amplified to 2–3 cm near the FOV edges (independent of edge location) due to susceptibility artifacts (up to ±0.1 mT/m shim gradients). For all systems, the spatial average chronic shim gradients exhibited notable temporal variability of 0.01–0.03 mT/m and were higher along SI (0.04–0.07) versus RL (0.01–0.04) (Supplemental Material S1). For Sys5 at SI offset locations, the 1st-order shim errors were the largest, with the chronic shim gradient ranging between –0.06–0.08 mT/m), with the corresponding spatial distortions varying linearly from −3 cm to +3 cm. These geometric distortions (data not shown) persisted for both SSE and DSE acquisitions, but apparently were not accompanied by substantial ADC bias (<3% for typical observed chronic gradient of 0.05 mT/m, Supplemental Material S1).

### 3.3. RMS Comparison of ADC Measurements

**Figure 4:**
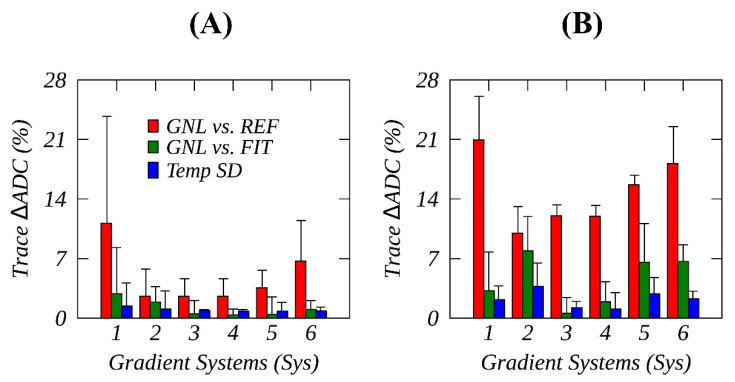
Root-mean-squared (RMS)% deviations (within ±18 cm), normalized by an ice-water apparent diffusion coefficient (ADC) reference (REF), are shown for predicted system model gradient nonlinearity (GNL) versus REF (red bars), GNL versus temporal mean fit (green bars), and temporal fit SD (blue bars) for trace ADCs, measured for horizontal offsets (**A**) and along the magnet bore (**B**) for six studied MR systems (Sys1-6, the error bars denote the largest RMS% observed among three physical gradient channels on a specific MR system, see Table 1).

The results of RMSD (%) analysis are summarized in Table 1 for individual gradients and the trace of six studied gradient systems (Sys1-6). The results for trace RMSD are also depicted in Figure 4 bar plots, along with the largest errors (error bars) among the three channels along SI and RL. On average, $\%RMSD_{REF}$ (Figure 4, red bars) was respectively 4.9 ± 3.2 (%) and 14.8 ± 3.8 (%) for measured RL and SI offsets; but increased to about 10% and 20% for Sys 1. This GNL bias was more than threefold higher than $\%RMSD_{EXP}$ (Figure 4, green bars) and fivefold higher than $\%RMSD_{TMP}$ (Figure 4, blue bars) for all systems except for Sys2, indicating that the spatial ADC bias could not be reliably measured for this specific gradient system.

On the other hand, the measured $\%RMSD_{EXP}$ (Figure 4, green bars) on average exceeded $\%RMSD_{TMP}$ (Figure 4, blue bars) for SI offsets, i.e., 4.5 ± 2.7 (%) vs. 2.2 ± 0.9 (%). These observations demonstrated that the measured spatial ADC biases was not fully accounted for by the system specific GNL models, particularly for Sys1 RL offsets and for Sys5 and Sys6 SI offsets (Table 1). These excessive deviations were partially rectified by DSE acquisition as previously demonstrated (Figure 3) for the diffusion gradient $G_{y}$ on Sys 1 and reduced $\%RMSD_{EXP}$ for $G_{z}$ of Sys 5 and Sys 6 (see Section 3.2). Generally, a higher temporal ADC variability observed for SI versus RL offsets (Table 1) concordant with measured shim results (Supplemental Material S1) suggested that shim gradients were likely contributors to the temporal ADC measurement errors.

Cross-system statistical metrics for SSE acquisition are summarized in Table 2. Absolute median GNL bias contribution was the largest (17%) for $G_{x}$ along SI, while $G_{z}$ was the most linear channel across systems along RL ($\%RMSD_{REF}$ < 2%). The observed RMSD ranges across systems confirmed GNL as the major source of cross-system variability ($\%RMSD_{REF}$, 10%–20% range for the different channels) versus systemic deviations from the model ($\%RMSD_{EXP}$, 2–6.5% ranges) and temporal measurement errors ($\%RMSD_{TMP}$, 0.5–3% ranges).

## 4. Discussion

This work summarized the analysis results from a two-year DWI stability evaluation using a temperature-controlled ice-water phantom to characterize the spatial and temporal ADC variations on six representative clinical MR scanners from three vendors. The ice-water provided universal reference ADC standard for evaluation of cross-system reproducibility, independent of the scanner room temperature. To complement previous phantom studies of the spatial ADC bias [10,12,17,19], this work included long-term longitudinal measurements to test the static character of the GNL relative to temporal variability and non-GNL bias sources over a large FOV typical for a range of body ADC applications [5,6,8]. The GNL models were provided by the MR vendors, the participants of the AIP. Additionally, the chronic shim gradients were also measured independently on all systems using $B_{0}$ mapping, and DSE variants were run when available to better differentiate between the non-GNL bias sources.

The present work confirmed the consistency and temporal stability of observed ADC bias with the theoretical prediction based on GNL-induced nonuniformity models, which are determined primarily by gradient system designs. Once built for a specific gradient system, the corrector maps can be applied retrospectively to the prior DWI scans of an arbitrary object and geometry [7,13]. Analogous to commonly used correction of geometric distortion, the prospective correction of GNL-induced DW bias can also be implemented on-scanner using a gradient system design and DWI scan geometry information [2,14].

Consistent with previous observations [10,12,17,19], uncorrected DW nonuniformity led to substantial errors both in absolute ADC values at offset locations and technical cross-system variability. One poorly performing gradient system that exhibited excessive measurement errors was not used for clinical trials but was included in this study for completeness. All other studied gradient systems manifested spatial ADC bias patterns consistent with their system specific GNL models (i.e., positive/negative biases with RL/SI offsets) for individual gradient channels. The highest absolute ADC deviations were observed along SI direction, reaching up to 30% spatial average error within an offset of ±18 cm. This ADC bias originated from GNL-induced nonuniformity in *b*-values and could be largely rectified as demonstrated before [1,2,3]. The observed differences between the measurements and theoretical models in excess of temporal variations indicated that other non-GNL sources contributed measurable systematic ADC biases.

One possible origin for the observed systematic deviations from GNL models could be imperfect *B_0_* shimming that introduced residual or chronic *B_0_* shim gradients [21] that were measured in this work. Our studies [23] with controlled chronic gradients demonstrated that the gradients observed in this study did not contribute to *b*-value errors by more than 5%. Furthermore, larger measured chronic gradients along SI correlated with the observation of higher temporal ADC variability, suggesting prevalent shim contribution to the temporal measurement errors.

Larger deviations from the GNL models (8–10%) observed for individual gradient channels of several systems were consistent with a non-shim, eddy current origin. Such systematic effects do not directly impact diffusion weighting [10], however it could cause an apparent spin density change primarily for DWI along a phase-encode direction [22]. Additionally, when present on a single channel, this cumulative bias is reduced for the direction-average ADC trace. These errors are also effectively mitigated by DSE, as was demonstrated in the current study. However, for thick axial slices, DSE may suffer from through-slice concomitant field dephasing, which may require additional correction [2]. These effects were not observed in the current work for a sagittal scan along SI. While retrospective correction of eddy current-induced spin density changes in ADC calculations for SSE is possible [25], it was outside of the focus of the model-based GNL correction.

Our results confirm that a constant residual shim gradient produces a proportional offset of diffusion weighting gradients (and *b*-value bias) [10], while an additional shim gradient slope leads to the spatial shift of the ADC bias curve along the applied DWI gradient direction [23]. This shift may increase the residual errors for locations far away from the isocenter. When DSE DWI is available, this shim-induced bias in *b*-value is empirically mitigated [10]. The observed geometric distortions were not reduced by DSE, which could lead to a spatial mismatch of the GNL correction map and anatomy location (up to 3 cm). These errors may be corrected by spatial registration to a non-EPI reference image. Alternatively, by measuring the *B_0_* map of the object, the presence of the residual shim gradients can be quantified and factored into an ideal GNL model by proportional spatial shifts and fractional offsets with respect to the isocenter.

The main limitation of this study is in providing only ADC and shim measurements along RL and SI lines that intersect the isocenter. This study design does not allow comprehensive 3D gradient system characterization and is not sensitive to the cross-channel GNL terms. The ADC measurements from the long-tube phantom geometry are restricted to one dimensional sparse spatial sampling. The cross-channel gradient contributions along sampled locations were inherently small due to spatial GNL symmetry [1,15], preventing reliable measurements. Other work using alternative phantoms and extensive 3D GNL mapping methods [14,17] have confirmed high consistency with the gradient model designs over moderate FOV. The current study workflow was chosen for an effective evaluation of temporal stability and relative static contribution of GNL and non-GNL bias sources over large FOV typical of body DWI. The advantage of using the ice-water phantom was in providing a single reference ADC for the evaluation of cross-system reproducibility, avoiding phantom diffusion dependence on scanner room temperature. Another advantage of the performed study was in applying the static system gradient models provided by vendors avoiding time complexity of the empiric gradient characterization that may introduce bias by a measurement method.

## 5. Conclusions

Gradient channel-specific ADC deviations from the reference (true) value were largely consistent with the static GNL model that well exceeded temporal variations for all scanners. This confirmed the feasibility of the effective correction of ADC bias based on the system GNL model both for retrospective and prospective correction approaches. Relative systematic deviations between measurements and GNL models that exceeded temporal variations were many-fold lower than GNL bias and more pronounced for individual gradient channels with eddy currents. The main cause for the deviations from GNL models was the intensity and geometric distortions from either local eddy currents or residual shim-gradients. The RMSD summary metrics applied in this study reasonably reflect observations that (a) GNL is the primary source of spatial ADC bias; (b) small additional systematic deviations from the GNL model exist due to shim and eddy currents; and (c) temporal variability is comparable to measurement uncertainty.

## 6. Patents

T. Chenevert and D. Malyarenko are the co-inventors of intellectual property assigned to and managed by the University of Michigan for the patented GNL-bias DWI correction technology (US9851426) licensed by Philips Medical Systems.

## Figures and Tables

**Table 1:** Summary of spatially averaged $\%RMSD_{i}$ (i=REF, EXP, TMP) for horizontal (RL) and along-the-bore (SI) offsets of three physical (*x*, *y*, *z*) gradient channels and the trace (*t*) from six studied gradient platforms (Sys1-6).

Sys	Grad	RL			SI		
		*REF*	*EXP*	*TMP*	*REF*	*EXP*	*TMP*
**1**	*x*	23.7	1.9	4.2	26.1	7.8	3.8
	*y*	8.7	8.3	1.3	24.7	4.2	2.3
	*z*	1.3	1.2	0.6	12.2	3.4	2.5
	*t*	11.2	2.9	1.4	20.9	3.2	2.2
**2**	*x*	5.8	3.7	3.2	13.0	5.6	2.5
	*y*	2.3	2.5	1.4	13.1	11.9	4.2
	*z*	0.4	2.5	1.1	3.9	7.0	6.5
	*t*	2.6	1.9	1.1	10.0	7.9	3.7
**3**	*x*	4.6	2.1	1.0	13.3	2.1	1.3
	*y*	2.1	0.7	1.0	13.2	2.4	1.1
	*z*	1.1	0.6	0.9	9.6	0.6	2.0
	*t*	2.6	0.5	0.9	12.0	0.6	1.2
**4**	*x*	4.6	1.1	1.0	13.2	1.5	0.9
	*y*	2.1	0.2	0.8	13.1	1.4	1.1
	*z*	1.1	0.2	0.8	9.6	4.3	3.0
	*t*	2.6	0.4	0.8	12.0	1.9	1.1
**5**	*x*	5.6	2.5	1.0	16.8	7.6	3.8
	*y*	3.7	0.7	0.5	16.8	4.1	3.3
	*z*	1.6	1.1	1.8	13.5	11.1	4.8
	*t*	3.6	0.5	0.8	15.7	6.6	2.9
**6**	*x*	11.5	2.1	1.2	22.5	5.1	1.5
	*y*	5.3	1.8	1.3	22.2	6.8	3.2
	*z*	3.3	1	0.4	9.7	8.6	2.6
	*t*	6.7	1	0.8	18.2	6.7	2.3

***REF***, reference, ***EXP***, measured; ***TMP***, temporal; **RL**, right to left; **SI**, superior to inferior; **1**–**6**, gradient system number; **RL**, right-left; **SI**, superior-inferior; **Sys**, system; **Grad**, gradient channel.

**Table 2:** Summary of cross-system variations (excluding Sys2) in spatial $\%RMSD_{i}$ (i=REF, EXP, TMP) of gradient-channel metrics listed in Table 1.

Grad		*x*			*y*			*z*			*t*		
RMSD		*REF*	*EXP*	*TMP*	*REF*	*EXP*	*TMP*	*REF*	*EXP*	*TMP*	*REF*	*EXP*	*TMP*
**RL**	Median	5.6	2.1	1	3.7	0.7	1	1.3	1	0.8	3.6	0.5	0.8
	Min	4.6	1.1	1	2.1	0.2	0.5	1.1	0.2	0.4	2.6	0.4	0.8
	Max	23.7	2.5	4.2	8.7	8.3	1.3	3.3	1.2	1.8	11.2	2.9	1.4
**SI**	Median	16.8	5.1	1.5	16.8	4.1	2.3	9.7	4.3	2.6	15.7	3.2	2.2
	Min	13.2	1.5	0.9	13.1	1.4	1.1	9.6	0.6	2	12	0.6	1.1
	Max	26.1	7.8	3.8	24.7	6.8	3.3	13.5	11.1	4.8	20.9	6.7	2.9

***REF***, reference, ***EXP***, measured; ***TMP***, temporal; **RL**, right to left; **SI**, superior to inferior; **Grad**, gradient channel.

## Data Availability

The data presented in this study are available on request from the corresponding authors.

## Supplementary Materials

The following supporting information can be downloaded at: https://www.mdpi.com/article/10.3390/tomography8010030/s1, Supplementary Material S1: Mean residual shim gradients from axial (red, RL) and sagittal (blue, SI) $B_{0}$ mapping. Supplementary Material S2: Icewater DWI Test Instructions.
