# Supplementary material for: Long-Term Stability of Gradient Characteristics Warrants Model-Based Correction of Diffusion Weighting Bias
Source: Tomography. 2022 Feb 4;8(1):364–75. doi: 10.3390/tomography8010030 (PMC8875771; doi:10.3390/tomography8010030)
Supplement: Supplementary file 1 [file tomography-08-00030-s001.zip › tomography-1567549-supplementary.pdf]

Supplementary Material (1)

Long-term stability of gradient characteristics warrants model-based correction of diffusion weighting bias

Yuxi Pang[1], Dariya I. Malyarenko[1*], Lisa J. Wilmes[2], Ajit Devaraj[3], Ek T. Tan[4], Luca Marinelli[5], Axel vom Endt[6], Johannes Peeters[7], Michael A. Jacobs[8], David C. Newitt[2] and Thomas L. Chenevert[1]*

[1]Radiology, University of Michigan, Ann Arbor, MI, United States; [2]Radiology and Biomedical Imaging, University of California San Francisco, San Francisco, CA, United States;[3]Philips Research Laboratories, Cambridge, MA, United States; [4]Radiology and Imaging, Hospital for Special Surgery, New York, NY, United States; [5]GE Global Research, Niskayuna, NY, United States; [6]Siemens Healthcare GmbH, Erlangen, Germany; [7]Philips MR Clinical Science, Best, Netherlands; [8]Radiology and Radiological Science, Johns Hopkins University School of Medicine, Baltimore, MD, United States

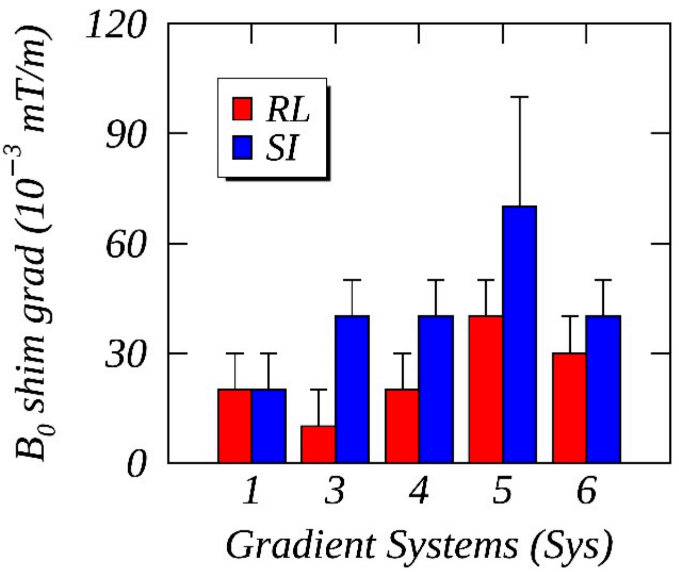

Supplementary Figure S1. Mean residual shim gradients (10^{-3} mT/m) estimated from axial (red, RL) and sagittal (blue, SI) B_0 mapping images acquitted at off-center positions for all gradient systems except Sys2. Error bars denote measurement standard deviations.

Supplementary Material (2)

ICEWATER DWI PHANTOM TEST INSTRUCTIONS
(DWI Gradient Non-Linearity Measurement)

EXPERIMENTAL OVERVIEW:

(1) *Phantom Preparation:* Prepare icewater phantom in advance of scanning (**SECTION 1**; *Fig.1a-c*)

(2) *Landmarking:* Prior to placing phantom, "landmark" on central lines scribed on the platform, and choose (adjustment bolt) hole locations so platform rests on the scan table and not the surface coil.

(3) *Platform Height Adjustment:* With phantom centered on platform and tube along **RL-axis**, perform survey scans for platform height adjustment to set tube center near 0mm along AP-axis (see **SECTION 2** and *Schema I a,b*)

(4) *DWI and B0 Shim Data*: Acquire DWI and B0 shim data with the phantom at each of six locations:
"Position 1" = phantom tube along RL-axis and phantom centered at RL=0mm
"Position 2" = phantom tube along RL-axis and phantom offset to Rt by 110mm
"Position 3" = phantom tube along RL-axis and phantom offset to Lt by 110mm
"Position 4" = phantom tube along SI-axis and phantom centered at SI=0mm
"Position 5" = phantom tube along SI-axis and phantom offset to Sup by 110mm
"Position 6" = phantom tube along SI-axis and phantom offset to Inf by 110mm

**** Note, only the phantom and "shim-box" are offset within the FOV ****
**** The FOV and acquired slices must remain centered at (0,0,0) mm ****

(5) *SNR Data:* Return phantom to "Position 4", and acquire two sequential DWI scans for NEMA-SNR

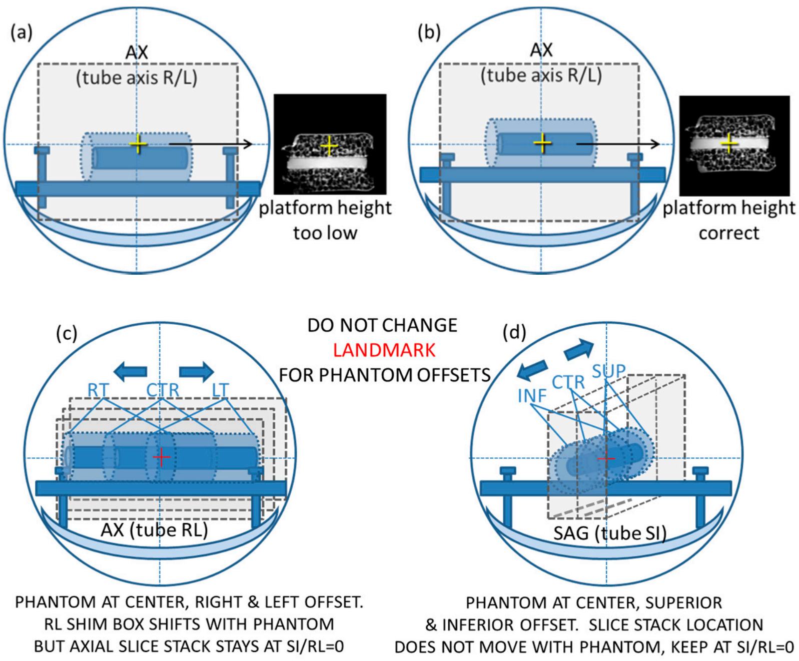

Schema I: Schematic of height adjustment such that center of tube is at AP=0 as achieved in (b) showing axial survey scan for RL tube axis orientation; (c) "Phantom Positions 1,2,3" RL offset AX scans; (d) Phantom "Positions 4,5,6" SI offset SAG scans.

SECTION 1. Icewater Phantom Preparation Procedure:

Please plan on filling the phantom with icewater well in advance of scanning. Once prepared, an icewater phantom requires approximately 1hour to reach thermal equilibrium. Once at equilibrium and in the foam insulation/plastic bag, the phantom will be usable for several (~4) hours. Keep the foam insulation sleeve and plastic bag shipped with the phantom for future use.

1) ***Contents:*** As shipped, the phantom consists of a 3.8 liter plastic container with a capped 175ml measurement tube pre-filled with distilled water, foam insulation sleeve, plastic bag and platform. The measurement tube is designed to be held in place by foam rings mounted to the top and bottom of the phantom. While the tube itself is not cemented in-place, it is preferred to leave the tube seated in the bottom foam ring. For ice-filling, temporarily remove the container from the foam insulation sleeve and plastic bag.

2) ***Air bubble:*** Visually inspect the measurement tube for air bubble while tipping the phantom over. A small air bubble (1-2cc's) is not a problem, but if much larger, please, add distilled water with a syringe following these steps: (a) remove the tube from the phantom; (b) remove the red cap from one end but leave the foam plug in place; (c) use a syringe to inject distilled water through the foam plug to displace the air bubble ; and (d) replace the red cap on the tube and seat the tube back in the phantom jar. If you have further questions about adding water, please, call/email Tom tlchenev@umich.edu 734-936-8866 or Dasha dariya@umich.edu at 734-647-5532 .

3) ***Initial ice fill:*** You will need a source of ice cubes or ice chips and a sink basin for phantom filling. The volume of ice cubes/chips required is approximately twice the volume of the phantom. Hold the measurement tube centered and seated in the phantom, and fill the phantom to the top with ice cubes/chips (*Fig.1a*). Add cold tap water and fill to the top (*Fig.1b*) – the colder the water, the better. Let the phantom sit for an "initial cool down period" of approximately 10 minutes.

4) ***Second ice fill:*** Depending on how cold your tap water is, much of the ice will melt relatively quickly. After the 10 minute initial cool-down period, pack-in as much ice as possible to replace the melted ice. Fill ice to the top allowing displaced water to overflow into the sink. The objective is to have ice cubes packed the full depth of the phantom and the interstitial space filled with water and minimal air (*Fig.1c*). Screw cap on tightly, dry off the phantom and inspect for leaks.

5) ***Set For Equilibrium:*** Put the phantom into the foam insulation sleeve with the container top toward the open end of the sleeve (*Fig.2a*), add the foam cap and put all in the plastic bag (*Fig.2b,c*). The plastic bag (in-place for scanning) will keep condensate off of MRI components. **Set the phantom aside for an additional 50minutes to allow the central tube to come to thermal equilibrium with the surrounding icewater.** As long as there is adequate ice in the phantom, temperature will be controlled to near 0°C thereby holding water within the tube at a known diffusion coefficient of 1.1x10^{-3}mm^{2}/s. The phantom should be usable for several hours (~3-4hrs) within the insulation sleeve. If desired, you can store the phantom in a refrigerator (not a freezer) to extend the usable time (5-10hrs).

6) ***After scanning:*** Empty the icewater down the drain but leave the central tube filled and sealed.

SECTION 2. Details of Phantom Positioning Procedure:

1) ***Landmarking (empty platform):*** Remove patient pads and place the platform on the scan table or the posterior half of the torso coil (*Figure 3a*). Depending on your MRI table width and coil width, the height-adjustment bolts may be moved to inward or outward openings to stabilize the platform so it rests on the table and not the coil.

   (a) Start with the platform set for lowest vertical height (in anterior/posterior AP direction) by unscrewing threaded "height-adjustment-bolts" so the platform is level but still supported by the bolts. For convenience, avoid having the bolts rest on a soft padded surface since we want the elevation of the platform predictably adjustable by the bolts. If needed, place a rubber cap (provided) between the bottom of each bolt and the padded surface.

   (b) Each height-adjustment-bolt has a nut on the top side of the platform. Finger-tighten these nuts so they bear down onto the top of the platform – to add stability to the platform rig (*Figure 4b*).

(c) Use the scanner alignment light to align the cross marked on the (empty) platform with alignment light crosshairs and "set patient landmark" on the center marked cross (*Figure 3a*). Make sure that the laser-cross is aligned with the center of the platform both RL and SI. (Adjust RL by physically moving the platform, if needed.) *For the remainder of the experiment, do not re-landmark or move the platform relative to landmark. This is to ensure that all slice stacks are centered at RL/SI = 0.*

2) ***Phantom Position 1 "Zero RL Offset":*** Position the phantom centered on the platform with measurement tube axis along RL direction inside the corner guides. The pink tape on the phantom should be aligned with the markings on the platform as shown in *Figure 3b*. Prop the tube end up very slightly so that air bubbles, if present, move toward the cap. Place anterior half of the torso coil on the phantom. If needed, use additional pads to support the sides of the anterior torso coil (*Figure 2c and Figure 3c*).

3) ***Platform Height Adjustment:*** Run a large (400x400mm) FOV sagittal or 3-orthogonal stacks survey. Measure the AP elevation of the center of the measurement tube using the scanner's electronic caliper, cursor-location, or ROI tool (*Schema I a*). Note the distance the center of measurement tube is below elevation "AP=0". The number of full turns to the height-adjustment-bolts will be this distance (in mm) divided by 2.

   (a) If you need to move the scan table out of the bore to gain access to the height-adjustment-bolts, do so with minimal movement of the platform on the table and without relandmarking. Loosen the 4 stabilization-nuts. To minimize movement of the platform, simply lift a corner of the platform with one hand while you screw in the height-adjustment-bolt the required number of clock-wise turns (one full turn = 2mm). Assuming the platform started out level, perform the same number of clockwise turns to each of the four height-adjustment-bolts. Inspect the (SI and RL) levels on the platform to ensure no substantial phantom tilt (*Figure 4a,b*). Re-finger-tighten the stabilization-nuts.

   (b) Re-run the survey to confirm the center of the measurement tube is at AP=0±5mm (*Schema Ib*). Tube may be slightly tilted so it may not be at AP=0±5mm along its full length, which is acceptable. Repeat height adjustment if necessary. When satisfied, perform DWI scans (and if possible B0 shim scan) for ***Phantom Position1 Zero RL Offset*** according to **Section3**. *Keep the AXIAL stack of slices centered at SI/RL=0 for all subsequent RL "Positions 2,3" and tailor shim volume to the phantom. DO NOT SHIFT THE AXIAL SLICE STACK.*

4) ***Phantom Position 2 "Right 110mm Offset":*** Temporarily remove the anterior torso coil, but do so with minimal movement of the platform on the table and without relandmarking. There should be no need to adjust the height of the platform. Simply reposition the phantom 110mm to "patient's right". The pink tape on the phantom should now align with the markings alongside the platform guides (*Figure 4c, Schema Ic*). Again, prop the phantom so the cap end is ever so slightly elevated to move bubbles to that end. Place the anterior torso coil on the phantom. Re-survey to document the phantom location and perform DWI (and B0 shim, if possible) scans according to **Section 3**.

5) ***Phantom Position 3 "Left 110mm Offset":*** Repeat Step 4, but with the phantom offset 110mm to "patient's left".

6) ***Phantom Position 4 "Zero SI Offset":*** Temporarily remove the anterior torso coil, but do so with minimal movement of the platform on the table and without relandmarking. There should be no need to adjust the height of the platform. Position the phantom so that the measurement tube runs along the SI direction. Align the tape stripe on the phantom with the marking running RL on the center of the platform (*Figure 5a, Schema Id*). Again, prop the phantom so the cap end is ever so slightly elevated to move bubbles to that end. Place the anterior torso coil on the phantom. Re-survey to document the phantom location and perform DWI (and if possible B0 shim) scanning according to **Section 3**. *For subsequent SI Positions 5,6*, *DO NOT SHIFT THE SAGITTAL SLICE STACK and only tailor shim volume to the phantom. The FOV and SAGITTAL stack of slices should stay centered at RL/SI = 0.*

7) ***<u>Phantom Position 5 "Superior 110mm Offset":</u>*** Temporarily remove the anterior torso coil, but do so with minimal movement of the platform on the table and without relandmarking. There should be no need to adjust the height of the platform. Simply reposition the phantom 110mm to "patient's superior". The pink tape on the phantom should now align with the marking alongside the platform guides (*Figure 5b*). Again, prop the phantom so the cap end is ever so slightly elevated to move bubbles to that end. Place the anterior torso coil on the phantom. Re-survey to document the phantom location and perform DWI (and if possible B0 shim) scanning according to **Section 3**.

8) ***<u>Phantom Position 6 "Inferior 110mm Offset":</u>*** Repeat Step 7, but with a 110mm offset to "patient's inferior" (*Figure 5c*).

SECTION 3. Details of DWI Icewater Phantom Scan Procedure:

Start a “New Patient/Exam” using “Head First, Supine” convention.

Overview of Series Required for DWI QC Phantom Scanning (for each “Position”):

1) ***Survey:*** three orthogonal stacks scout using a large (400x400mm) FOV *after each phantom repositioning.*
2) ***SSE DWI and shim map (phantom along RL):*** *For each of phantom Position 1, 2, and 3*, acquire 15 AXIAL slices with DWI along X,Y,Z LAB axes using single-shot echo-planar-imaging (EPI) at b-values = 0, 750 and 1500 s/mm^2 (with single spin-echo SSE sequence). Save co-registered DWI series and acquire B0 shim map. For each “Position 1, 2, 3” also The FOV is intentionally much larger than needed to cover the phantom. This is so that the location of the AXIAL DWI slice stack IS NOT CHANGED for phantom Positions 1, 2 and 3.
    a. *Move ONLY the shim volume with the phantom, but KEEP the FOV and AXIAL stack of slices centered at SI/RL=0.* **The phantom is repositioned within the fixed stack of slices. It is acceptable (preferred) to keep the shim-volume centered on phantom regardless of its location, but keep center of the slice stack fixed at isocenter.**
    b. **BE SURE EACH LAB AXIS DWI IS GENERATED** – NOT JUST THE TRACE DWI!!! That is, for each slice generate DWI_0; DWI_{750X}; DWI_{750Y}; DWI_{750Z}; DWI_{1500X}; DWI_{1500Y} ; DWI_{1500Z}. Production of isotropic/trace DWI is optional. Save co-registered series.
    c. If possible, acquire a B0 (shim) map for a single (center slice) using your system-specific method (e.g., multi-echo GRE producing complex images).
3) ***SSE and DSE DWI, and shim map (phantom along SI):*** *For each of phantom Position 4, 5, and 6*, acquire 15 SAGITTAL slices with DWI along X,Y,Z LAB axes using single-shot echo-planar-imaging (EPI) at b-values = 0, 750 and 1500 s/mm^2 (with single and double spin echo sequences.) Save co-registered DWI series and acquire B0 shim map. The FOV is intentionally much larger than needed to cover the phantom. This is so that the location of the SAGITTAL DWI slice stack IS NOT CHANDED for phantom Positions 4, 5, 6.
    a. *Move ONLY the shim volume with the phantom, but KEEP the FOV and SAGITTAL stack of slices centered at SI/RL=0.* **The phantom is repositioned within the fixed stack of slices. It is acceptable (preferred) to keep the shim-volume centered on phantom regardless of its location, but keep center of the slice stack fixed at isocenter.**
    b. **BE SURE EACH LAB AXIS DWI IS GENERATED** – NOT JUST THE TRACE DWI!!! That is, for each slice generate DWI_0; DWI_{750X}; DWI_{750Y}; DWI_{750Z}; DWI_{1500X}; DWI_{1500Y} ; DWI_{1500Z}. Save co-registered series. Production of isotropic/trace DWI is optional.
    c. If your system is capable of twice-refocused SE, perform additional double spin-echo DWI measurements for all SI positions.
    d. If possible, acquire a B0 (shim) map for a single (center slice) using your system-specific method (e.g., multi-echo GRE producing complex images).
4) ***SNR DWI scan:*** Return phantom to “Position 4” to perform two consecutive SSE DWI acquisitions (for NEMA-SNR estimation) with 5 AXIAL slices (5mm skip 5mm).

Table 1. DWI Icewater Phantom Scan Parameters				
Field Strength	1.5T or 3T		DWI Sequence	Single-Shot SE EPI Single Echo (SSE) and Double Echo (DSE)
Receiver Coil	Torso Array		TR (ms)	≥10000
FOV (mm)	380 x 380		TE (ms)	min or ≤150
Acquisition Matrix	200 x 200 [a]		Half-scan, Partial-Fourier, Frac-NEX	No [a]
Reconstruction Matrix	256 x 256 [b]		Number of Gradient Directions [c]	3 LAB axes
Orientation	True Axial, no obliquity (Foldover, Phase AP; Freq. RL) for "Position 1,2,3"; True Sagittal (Phase AP, Freq. SI) for "Position 4,5,6")		Freq Enc Bandwidth per Acq Pixel (Hz) [d]	1500 to 2500
Quantity of Slices	15 slices for phantom "Positions 1-6"<br><br>5 slices for phantom position 4 (SNR scan)		Parallel Imaging	NO
Slice Thickness (mm)	5		b-values (s/mm^2) [e]	0, 750, 1500
Gap (mm)	0 for phantom "Positions 1-6"<br><br>5 for phantom "Positions 4" (SNR scan)		# Signal Averages	2
Image Filtering (eg.SCIC or CLEAR)	Off (Classic)		Fat Suppression [f]	No

a) Some systems may require "half-scan", "partial-Fourier", or "fractional-NEX" for DWI. It is preferred to acquire the full phase-encode matrix = 160 by setting half-scan/ partial-Fourier/Fractional-NEX to "No" or at least as close to 1 as possible.

b) Interpolate image matrix to 256 x 256. Some systems may do this automatically.

c) Acquire DWI along three orthogonal LAB axes so that direction specific DWIs are generated. Isotropic or trace diffusion weighted images are optional.

d) Frequency encoding bandwidth may not be under full operator control. If possible use "maximum bandwidth", or equivalently "minimum fat shift per pixel", or set within 1500 to 2500 Hz/pixel range.

e) It is preferred to acquire all three b-values in a single series.

f) The phantom does not contain fat signal, so fat suppression is optional, however DO NOT USE the Inversion-Recovery (STIR) fat suppression.

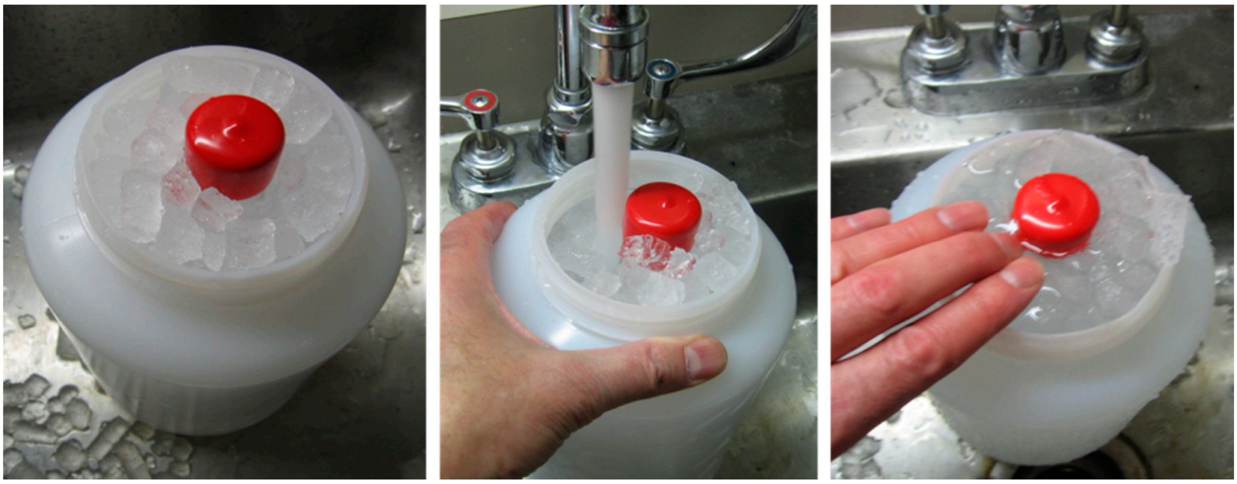

Figure 1: (a) (b) (c)

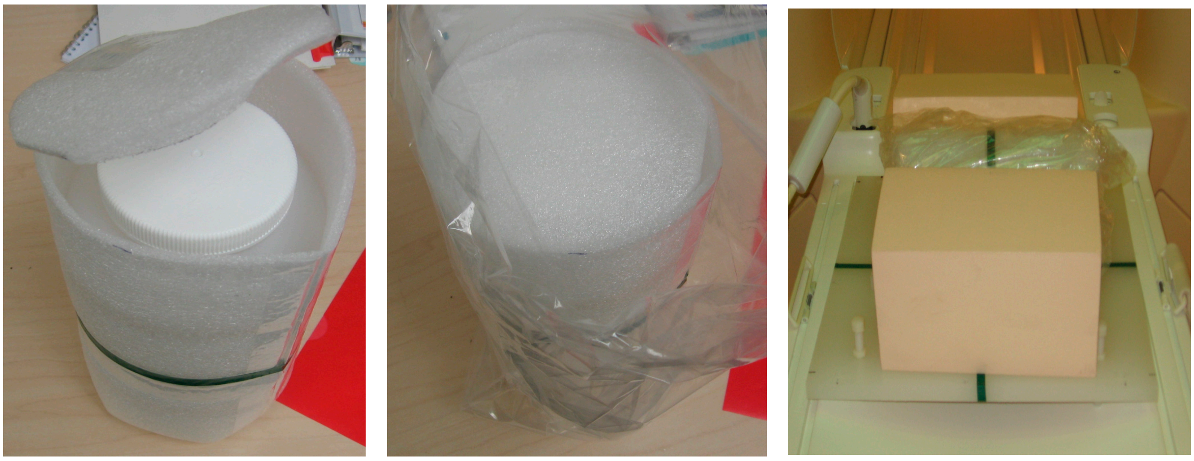

Figure 2: (a) (b) (c)

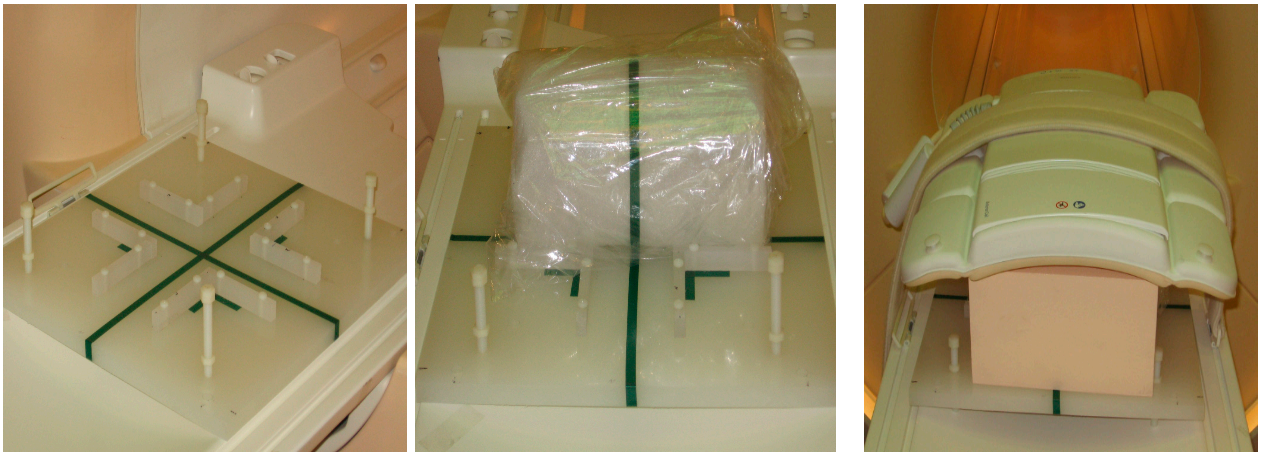

Figure 3: (a) (b) (c)

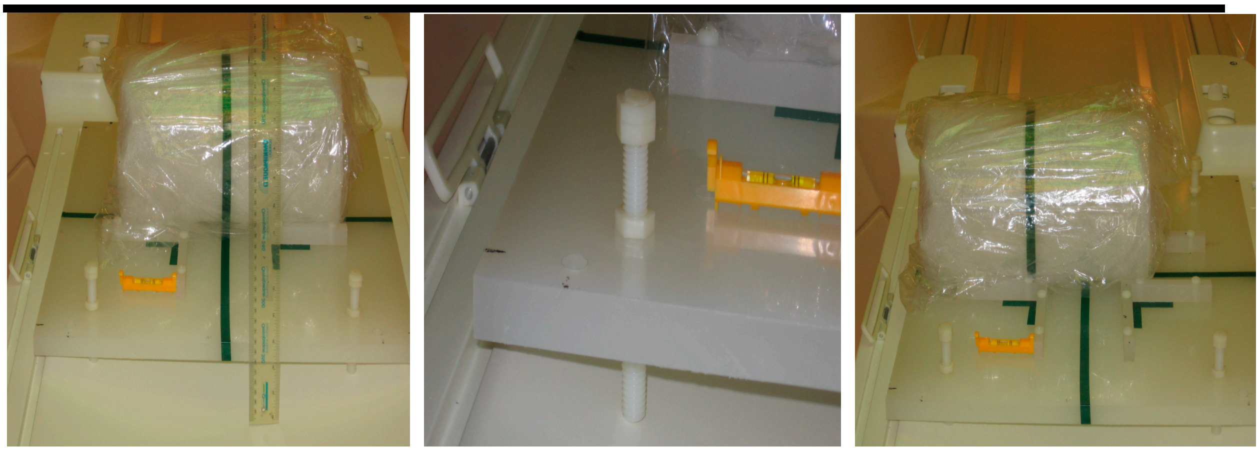

Figure 4: (a) (b) (c)

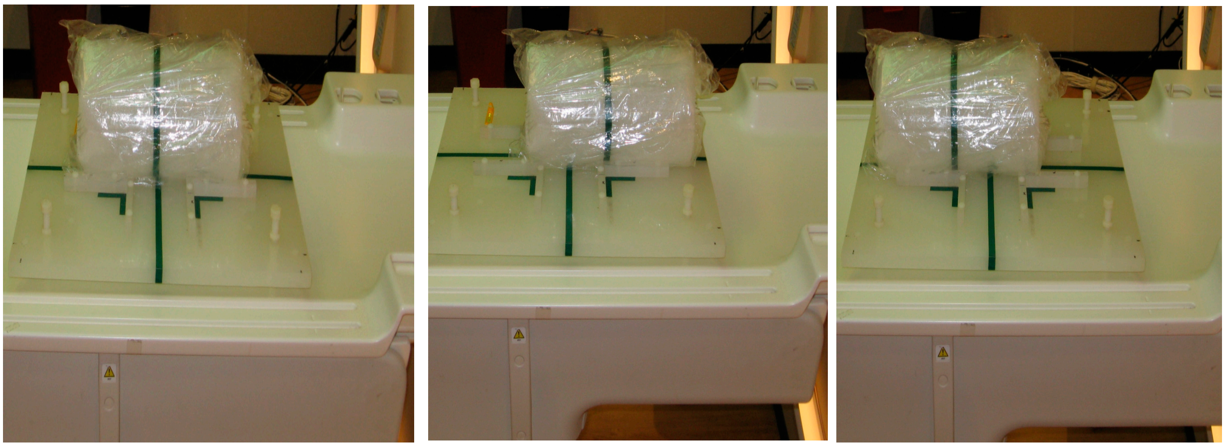

Figure 5: (a) (b) (c)
